# Neuropsychological assessment in MS is outdated and in need for innovation: NO

**DOI:** 10.1177/13524585241227773

**Published:** 2024-01-30

**Authors:** Dawn Langdon

**Affiliations:** Royal Holloway University of London, Egham, UK

The widespread negative impacts of MS cognitive impairments, most tellingly on disease management, relationships and employment, mean that assessment remains an essential component of clinical care. Importantly, it has direct benefits for people with MS (PwMS).^1^ It can also signal the importance to PwMS of adopting the Brain Health agenda. It may serve as a flag for breakthrough disease and initiate assessments that indicate treatment escalation. It allows health care professionals to accommodate the cognitive profile of the PwMS and amend their clinical interactions and support appropriately.^2^ It can triage PwMS for cognitive rehabilitation, targeting scarce resources. In the research context, it allows monitoring of cognitive outcomes in treatment and rehabilitation trials. The international MS community, whether represented by professional organisations, government bodies, expert panels, or specialist centres, has a consensus view that objective neuropsychological assessment should be offered on an annual basis in MS clinics.^2^

What alternatives do we have to formal neuropsychological assessment? Some would argue that magnetic resonance imaging (MRI) measures cognition. In fact, brain T2 lesion load only correlates 0.3 with objective cognitive performance.^3^ Even brain atrophy, not available in most clinical contexts, only manages a 0.5 correlation. More research-based magnetic resonance (MR) sequences, such as resting state fMRI, have an ambivalent relation to cognitive function.^4^ Asking PwMS about their cognitive competence, although essential for clinical care, does not align with objective neuropsychological assessment.^5^ Self-report is heavily influenced by mood and other psychosocial factors. Relatives of PwMS do rather better when reporting their loved one’s cognitive competence, but they may not always be available at consultations. Health care professionals are poor at determining the cognitive status of PwMS, when they rely on routine clinic consultations.

One reason for seeking a replacement to traditional neuropsychological assessment in routine MS clinical care is inadequate resources, with the suggestion that painstaking and expensive one-on-one neuropsychological assessments belong to a bygone age when health services were better funded. The Brief International Cognitive Assessment for MS (BICAMS) reduces the resource requirement significantly. It is a 15-minute assessment, requiring little specialist equipment and can be administered by most health professionals. It is now validated in 26 countries.^6^ It is in widespread research use, and to a lesser extent clinical use, across the globe. It does not provide a detailed and extensive cognitive profile, but it is psychometrically robust when determining if cognitive impairment is present. For clinics with very little time to devote to cognitive assessment, the Symbol Digit Modalities Test given alone is worth the 5 minutes taken. More extensive MS validated neuropsychological batteries are available for those centres with better resourcing.^7^ The most established are the Brief Repeatable Battery of Neuropsychological Tests (BRBN) and the Minimal Assessment for Cognitive Function in MS (MACFIMS), taking 45 and 90 minutes, respectively.

It can be fairly argued that digital health formats are overdue for widespread adoption, both generally and for MS neuropsychological assessment. It is likely that digital capture of cognitive performance will reduce the requirement for health professional expertise when assessing PwMS cognition. Smartphones are fairly ubiquitous around the world and would seem the obvious platform. However the confounds of MS distractibility and reduced dual-tasking make the uncontrolled testing context influential on results. Also the PwMS/phone interface remains dogged by software, hardware and sensorimotor variability. Attrition remains a major stumbling block.^8^ The same interface quandries arise with computerised testing, at home or in the clinic, although some assessment programmes are building psychometric credibility.^9^ Virtual reality paradigms offer more ecological validity and sensitivity to how cognitive difficulties play out in everyday life.^10^ But the construction of equivalent tasks across different cultures within, let alone between, countries is difficult. The need to assess information processing speed, almost certainly involving timed performance, makes digital assessment vulnerable to hardware and software variations. These issues may all reduce the validity of the data captured. The feasibility and relative expense of the required digital technology and infrastructure for any of these options will likely result in patchy provision. This risks exacerbating health inequalities, already implicated in MS health outcomes.

Current improvements in neuropsychological assessment technologies are to be welcomed, and we should continue to strive energetically for more. However, far greater and immediate benefits for PwMS could be achieved by implementing an international cognition management protocol. This could involve best available current neuropsychological assessment (depending on local resource level and staff expertise); careful preparation of PwMS and their loved ones for the assessment and feedback on the results; clinic staff adopting appropriate interaction styles for all disease management and monitoring any increased risks resulting from the documented cognitive impairment profile; discussion and supportive implementation of Brain Health; provision of/referral to more detailed cognitive assessment and rehabilitation; and full engagement of PwMS, carers and their systems to ensure understanding of their cognitive status, facilitating best QoL and health outcomes.^2^ It is our failure to address cognition which is truly outdated and the pressing need for innovation lies in provision of effective cognition assessment and management.

## References

[1] Longley WA Tate RL Brown RF . The psychological benefits of neuropsychological assessment feedback as a psycho-educational therapeutic intervention: A randomized-controlled trial with cross-over in multiple sclerosis. Neuropsychol Rehabil 2023; 33(5): 764–793. 35332853 10.1080/09602011.2022.2047734

[2] Langdon D Young CA . Fast facts: Cognition in multiple sclerosis. Karger, 2023, https://karger.com/books/book/3562/Fast-Facts-Cognition-in-Multiple-Sclerosis

[3] Mollison D Sellar R Bastin M , et al. The clinico-radiological paradox of cognitive function and MRI burden of white matter lesions in people with multiple sclerosis: A systematic review and meta-analysis. PLoS ONE 2017; 12(5): e0177727. 10.1371/journal.pone.0177727 PMC5432109 28505177

[4] Jandric D Doshi A Scott R , et al. A Systematic review of resting-state functional mri connectivity changes and cognitive impairment in multiple sclerosis. Brain Connect 2022; 12(2): 112–133. 34382408 10.1089/brain.2021.0104

[5] Tunmore J Kontou E Moghaddam N , et al. The association between the Multiple Sclerosis Screening Questionnaire and objective measures of cognition: A systematic literature review and meta-analysis. J Clin Exp Neuropsychol 2023; 45(2): 197–217. 37272878 10.1080/13803395.2023.2213847

[6] Potticary H Langdon D . A systematic review and meta-analysis of the brief cognitive assessment for multiple sclerosis (BICAMS) international validations. J Clin Med 2023; 12(2): 703. 36675637 10.3390/jcm12020703 PMC9863826

[7] Elwick H Topcu G Allen CM , et al. Cognitive measures used in adults with multiple sclerosis: A systematic review. Neuropsychol Rehabil 2022; 32(9): 2464–2481. 34121613 10.1080/09602011.2021.1936080

[8] Foong YC Bridge F Merlo D , et al. Smartphone monitoring of cognition in people with multiple sclerosis: A systematic review. Mult Scler Relat Disord 2023; 73: 104674. 37001409 10.1016/j.msard.2023.104674

[9] Wojcik CM Beier M Costello K , et al. Computerized neuropsychological assessment devices in multiple sclerosis: A systematic review. Mult Scler 2019; 25(14): 1848–1869. 31637963 10.1177/1352458519879094 PMC6875828

[10] Hsu WY Goverover Y Bove RM . Capturing cognitive changes in multiple sclerosis by performance-based functional and virtual reality assessments. Ann Phys Rehabil Med 2023; 66(3): 101677. 35667625 10.1016/j.rehab.2022.101677

